# Hydroxychloroquine Use in Patients With COVID-19: A Brief Perspective on Current Clinical Trials

**DOI:** 10.31486/toj.20.0124

**Published:** 2020

**Authors:** Alaa A. Abd-Elsayed, David Hao, Robert Chu, Ivan Urits, Omar Viswanath, Vwaire Orhurhu

**Affiliations:** ^1^ Department of Anesthesiology, Division of Pain Medicine, University of Wisconsin School of Medicine and Public Health, Madison, WI; ^2^ Department of Anesthesia, Critical Care and Pain Medicine, Massachusetts General Hospital, Harvard Medical School, Boston, MA; ^3^ Johns Hopkins School of Medicine, Baltimore, MD; ^4^ Department of Anesthesia, Critical Care and Pain Medicine, Beth Israel Deaconess Medical Center, Harvard Medical School, Boston, MA; ^5^ Department of Anesthesiology, Creighton University School of Medicine, Omaha, NE; Valley Anesthesiology and Pain Consultants, Envision Physician Services, Phoenix, AZ; and Department of Anesthesiology, University of Arizona College of Medicine-Phoenix, Phoenix, AZ

## INTRODUCTION

Severe acute respiratory syndrome coronavirus 2 (SARS-CoV-2), the virus responsible for coronavirus disease 2019 (COVID-19), remains elusive and nonresponsive to medication management. As the medical community forges ahead with exploring therapies, we must understand and learn from the clinical trials that have investigated the use of hydroxychloroquine (HCQ) and chloroquine (CQ) in patients with COVID-19.

Initial interest in the use of HCQ was triggered by a small nonrandomized study that has since received heavy criticism for both its statistical methods and potential conflicts of interest, as the journal editor-in-chief was included as an author.^1-3^ As a result of this controversial study and the subsequent widespread discussion of HCQ in the lay media, rigorous study of HCQ and CQ use in patients with COVID-19 was needed.

## OVERVIEW OF CLINICAL TRIALS

Since the first case of COVID-19 was reported in December 2019, a total of 10 randomized clinical trials (RCTs) have investigated treatment with HCQ (Table).^4-13^ These clinical trials included patients at multiple stages of severity, including asymptomatic without hospitalization, symptomatic with mild or moderate illness, and symptomatic with severe illness. From the outset, the external validity of studies investigating HCQ use in patients with COVID-19 has been challenged by heterogeneous methods of patient selection that have ranged from asymptomatic individuals with an identified exposure to hospitalized patients with clinical suspicion to positive reverse transcriptase–polymerase chain reaction (RT-PCR) with chest computed tomography (CT) evidence of pneumonia. In one RCT, methods of diagnostic confirmation of COVID-19 were not even specified.^4^ Drawing conclusions from highly variable clinical and laboratory diagnostic methodologies is fraught with potential error. Irrespective of the ultimate conclusions of the studies, we start first with the appreciation that not all studies are created equal. To add to the variability of the trials, the intervention and treatment dosing fluctuated substantially from center to center. Although the majority of studies elected to compare HCQ to standard of care, the dose and duration of HCQ treatment in the experimental group and what constituted standard of care varied significantly among studies. To add perspective, only 2 trials had an identical intervention regimen of 400 mg HCQ for a cumulative 5 days.^4,5^ With these caveats in mind, we turn to the outcomes of these trials.

**Table.** Characteristics of Clinical Trials Investigating Hydroxychloroquine Use in Patients With Coronavirus Disease

	Study and Publication Date				
Study Characteristic	Borba et al, 2020^11^	Boulware et al, 2020^6^	Cavalcanti et al, 2020^7^	Chen CP et al, 2020^10^	Chen J et al, 2020^4^
Study design	Double-blind, randomized controlled	Double-blind, randomized controlled	Multicenter, open-label, randomized controlled	Multicenter, open-label, randomized controlled	Randomized controlled
Country/countries of origin	Brazil	United States and Canada	Brazil	Taiwan	China
Patient population	Hospitalized patients ≥18 years, clinical suspicion of COVID-19 with ≥1 of respiratory rate >24/min, heart rate >125/min, O_2_ saturation <90% on room air, shock	Asymptomatic individuals ≥18 years, household or occupational exposure to a person with confirmed COVID-19 at <6 feet for >10 minutes	Hospitalized patients ≥18 years, suspected or confirmed COVID-19 within 14 days of symptom onset	Patients 20-79 years, mild or moderate illness by CXR	Patients ≥18 years, confirmed moderate COVID-19 disease
Method of COVID-19 testing	RT-PCR (patients were enrolled before results returned)	None for study subjects Contacts initially only required to have presumptive COVID-19; switched to RT-PCR confirmation during enrollment	RT-PCR	RT-PCR	Not specified
Intervention	High-dose CQ + standard of care (n=81)	HCQ (n=414)	HCQ + standard of care (n=59) HCQ + azithromycin + standard of care (n=172)	HCQ + standard of care (n=21)	HCQ + standard of care (n=15)
Treatment medication dose	CQ 600 mg twice daily for 10 days Ceftriaxone 1 g twice daily for 7 days Azithromycin 500 mg daily for 5 days	800 mg once, then 600 mg 6-8 hours later, then 600 mg daily for 4 days	HCQ 400 mg twice daily for 7 days Azithromycin 500 mg daily for 7 days + standard of care	HCQ 400 mg twice daily on day 1, then 200 mg twice daily for 6 days + standard of care	HCQ 400 mg daily for 5 days + standard of care
Control	Low-dose CQ + standard of care (n=40)	Placebo (n=407)	Standard of care (n=173)	Standard of care (n=12)	Standard of care (n=15)
Control medication dose	CQ 450 mg twice daily on day 1, then 450 mg daily for 4 days Ceftriaxone 1 g twice daily for 7 days Azithromycin 500 mg daily for 5 days	N/A	Not specified; at discretion of treating physicians Glucocorticoids, immunomodulators, antibiotics, and antivirals permitted	Ceftriaxone 2 g daily for 7 days ± azithromycin 500 mg on day 1, then 250 mg for 4 days OR levofloxacin 750 mg daily for 5 days OR levofloxacin 500 mg daily OR moxifloxacin 400 mg daily for 7-14 days	Not specified; included interferon-α, umifenovir, lopinavir, ritonavir, antibiotics
Primary endpoints	Mortality by day 28	Symptomatic illness with positive molecular assay or COVID-19–related symptoms	Clinical status at 15 days	Time to negative RT-PCR from randomization until 14 days	Proportion of negative SARS-CoV-2 pharyngeal swabs on day 7
Secondary endpoints	Mortality by day 13 Participant clinical status and laboratory examinations ECG on days 13 and 28 Duration of supplemental oxygen and/or mechanical ventilation Time from treatment initiation to death	Hospitalization or death from COVID-19 Discontinuation of intervention for any cause Symptoms at days 5 and 14	Clinical status at 7 days Intubation within 15 days HFNC or noninvasive ventilation use within 15 days Duration of hospital stay In-hospital mortality Thromboembolism AKI Days alive and free of respiratory support up to 15 days	Proportion of negative viral RT-PCR on day 14 Resolution of clinical symptoms Proportion discharged by day 14 Mortality rate	Mortality Time to negative seroconversion Time to body temperature normalization Radiologic evidence of improvement Safety profile
Results	Study stopped after interim analysis showed increased incidence of QTc interval prolongation and lethality in high-dose CQ group Because of small sample size, unable to show any benefit regarding treatment efficacy	No significant difference in development of new COVID-19 No mortality	No significant difference in clinical status at day 15 No significant difference in any secondary outcome	No significant difference in time to negative RT-PCR No significant difference in proportion of negative RT-PCR on day 14 No mortality in either group	No significant difference in proportion of negative testing at 7 days No significant difference in time to negative seroconversion, time to body temperature normalization, or radiologic improvement No mortality
Study limitations	Small sample size Single center No placebo group No exclusion criteria based on baseline QTc No chest CT scans to evaluate disease severity More patients with heart disease assigned to high-dose vs low-dose group High-dose group more susceptible to cardiac complications with or without medication use	Single center Most subjects were health care workers Most subjects unable to access COVID-19 testing to confirm infection Online recruitment led to generally younger and healthier research population (selection bias) and increased difficulty of follow-up Exposure poorly defined No outcomes based on imaging or viral load Moderate adherence	Large range of odds ratios Unblinded Protocol deviations related to lack of medication Included patients who had received HCQ and/or azithromycin before study enrollment Included patients up to 14 days after beginning of symptoms Unspecified control group treatment No outcomes based on imaging or viral load Fewer patients in control group had serial ECGs during follow-up	Only included patients with mild/moderate illness Small sample size Younger patient population (mean 32.9 years) than other studies Three patients concomitantly administered azithromycin No outcomes based on imaging	Single center Only included patients with moderate disease No diagnosis confirmation method specified Small sample size Heterogeneous control group treatment, including antivirals and unspecified antibiotics No QTc monitoring
Medication-related adverse events	QTc prolongation (>500 ms with CQ) Myopathy and rhabdomyolysis with CQ Myocarditis related to SARS-CoV-2 may progress to arrhythmias with prolonged QTc	Increased incidence of adverse events in HCQ group; most commonly GI upset, neurologic symptoms (headache, vertigo, irritability) No cardiac arrhythmias or other serious complications	Increased incidence of adverse events in HCQ group Increased risk of QTc prolongation in HCQ group Increased risk of aminotransferase elevation in HCQ + azithromycin vs control groups	HCQ-associated GI upset, headache, dizziness No QTc prolongation reported	No difference in adverse events between groups Mild diarrhea, muscle weakness, and ALT elevations reported in HCQ group
	**Study and Publication Date**				
**Study Characteristic**	**Chen L et al, 2020**^8^	**Chen Z et al, 2020**^5^	**Mitjà et al, 2020**^12^	**Skipper et al, 2020**^13^	**Tang et al, 2020**^9^
Study design	Open-label, randomized, controlled	Double-blind, randomized controlled	Multicenter, open-label, randomized controlled	Multicenter, double-blind, randomized controlled	Multicenter, open-label, randomized controlled
Country/countries of origin	China	China	Spain	United States and Canada	China
Patient population	Patients 18-75 years, mild or moderate illness	Patients ≥18 years positive for SARS-CoV-2 by RT-PCR, mild illness (SaO_2_/SpO_2_ >93% or PaO_2_/FiO_2_ >300), chest CT evidence of pneumonia	Nonhospitalized patients ≥18 years, mild symptoms of COVID-19	Nonhospitalized patients ≥18 years, <4 days of COVID-compatible symptoms	Patients ≥18 years positive for SARS-CoV-2 by RT-PCR
Method of COVID-19 testing	RT-PCR or chest CT	RT-PCR	RT-PCR	PCR-confirmed COVID-19 or exposure to individual with PCR-confirmed COVID-19 within 14 days	RT-PCR
Intervention	HCQ + standard of care (n=18) CQ + standard of care (n=18)	HCQ + standard of care (n=31)	HCQ + standard of care (n=136)	HCQ + standard of care (n=212)	HCQ + standard of care (n=75)
Treatment medication dose	HCQ 200 mg twice daily for 10 days CQ 1,000 mg on day 1, then 500 mg daily for 9 days + standard of care	HCQ 400 mg daily for 5 days	HCQ 800 mg on day 1, then 400 mg daily for 6 days	HCQ 800 mg once, then 600 mg 6-8 hours later, then 600 mg once daily for 4 days	HCQ 1,200 mg daily for 3 days followed by 800 mg daily for 2 weeks for mild-moderate disease, 3 weeks for severe disease
Control	Standard of care (n=12)	Standard of care (n=31)	Standard of care (n=157)	Placebo (n=211)	Standard of care (n=75)
Control medication dose	Not specified	Not specified; included antivirals, antibiotics, immunoglobulins, corticosteroids	Not specified	N/A	Not specified; according to Chinese national guidelines
Primary endpoints	Time to clinical recovery	Time to clinical recovery	Reduction of viral RNA load on days 3 and 7	Clinical outcome at day 14	Proportion of negative SARS-CoV-2 by 28 days
Secondary endpoints	Time to negative RT-PCR Length of hospital stay Changes on chest CT Duration of supplemental oxygen Frequency of adverse events Clinical status All-cause mortality Vital signs Inflammatory marker testing	Radiologic evidence of improvement Safety profile	Clinical progression Time from randomization to resolution of symptoms Safety profile	Symptom severity at days 5 and 14 Incidence of hospitalization or death Incidence of study medicine withdrawal	Clinical improvement Inflammatory marker levels Radiologic evidence of improvement Mortality Adverse events
Results	Shorter time to clinical recovery in CQ group and trend toward shorter recovery in HCQ group Shorter time to negative RT-PCR in both CQ and HCQ groups Trend toward decreased hospitalization length and improved CT chest changes in both CQ and HCQ groups No mortality in any group No significant difference in inflammatory markers	Significantly quicker time to clinical recovery in HCQ group Increased improvement in chest CT (80.6% vs 54.8%) in HCQ group	No significant difference in viral RNA load at days 3 or 7 No significant difference in hospitalization or time to symptom resolution No mortality	No significant difference in symptom resolution, hospitalization, or death between treatment groups	No significant difference in proportion of negative conversion or speed to negative conversion No difference in clinical course No difference in inflammatory markers No mortality
Study limitations	Single center Unblinded Only included patients with mild/moderate illness Small sample size HCQ dosing based on rheumatoid arthritis treatment Unspecified control group treatment	Single center Only included patients with mild illness Small sample size Heterogeneous control group treatment Relatively short observation period No outcomes based on viral load No QTc monitoring	Unblinded Only included patients with mild illness Standard of care treatment not specified No initial plan to evaluate clinical assessments on day 7, so fewer results than on day 3 High (>80%) enrollment of health care workers No outcomes based on imaging No QTc monitoring	Majority of subjects were health care workers Underrepresentation of Black/African American subjects Lack of laboratory-confirmed SARS-CoV-2 infection in all participants Enrollment of patients based on epidemiologic links to known cases No outcomes based on imaging or viral load Moderate adherence No QTc monitoring	Unblinded High proportion (98%) of patients with mild/moderate disease Relatively long lead-in time from symptom onset to treatment (16 days) Premature termination of enrollment for lack of participants
Medication-related adverse events	Increased incidence of mild adverse events in HCQ and CQ groups, most commonly diarrhea, nausea, ALT/AST elevation One grade 2 ALT/AST elevation in HCQ group No cardiac adverse events reported	Two patients in HCQ group with mild adverse events (rash and headache) No severe adverse events reported	Increased incidence of adverse events in HCQ group (GI upset, drowsiness, headache, taste change) No HCQ-related serious adverse events	Increased incidence of adverse events in HCQ group (GI upset) No serious adverse events attributable to HCQ	Increased incidence of adverse events in HCQ patients (GI upset, blurry vision, increased thirst) Two serious adverse events in HCQ group (disease progression, upper respiratory tract infection) No cardiac arrhythmias reported

AKI, acute kidney injury; ALT, alanine aminotransferase; AST, aspartate aminotransferase; COVID-19, coronavirus disease 2019; CQ, chloroquine; CT, computed tomography; CXR, chest x-ray; ECG, electrocardiogram; FiO_2,_ fraction of inspired oxygen; GI, gastrointestinal; HCQ, hydroxychloroquine; HFNC, high-flow nasal cannula; N/A, not applicable; O_2_, oxygen; PaO_2,_ partial pressure of arterial oxygen; PCR, polymerase chain reaction; RNA, ribonucleic acid; RT-PCR, reverse transcription polymerase chain reaction; SaO_2,_ oxygen saturation on arterial blood gas; SARS-CoV-2, severe acute respiratory syndrome coronavirus 2; SpO_2,_ oxygen saturation by pulse oximetry.

The largest study as of October 2020 (n=821) observed development of positive molecular assay or COVID-19–related symptoms in previously asymptomatic individuals with exposure to confirmed COVID-19.^6^ No significant difference was observed between the HCQ treatment group (one-time 800-mg dose followed by 600 mg/day for 5 days) and placebo group in the development of COVID-19, with the notable caveat that the majority of participants had limited access to COVID-19 testing.

An open-label RCT among hospitalized patients with COVID-19 used a regimen of HCQ (400 mg twice daily) plus standard of care or HCQ with azithromycin (500 mg daily) plus standard of care. Results indicated no significant differences in the primary outcome of clinical status at day 15 or any secondary outcomes, including use of noninvasive ventilation, in-hospital mortality, or duration of hospital stay.^7^ Two relatively large (n>100) RCTs also failed to demonstrate improvement in viral parameters (viral load) or clinical outcomes (hospitalization, mortality, symptom resolution) in nonhospitalized patients treated with HCQ compared to standard of care or placebo.^12,13^

As we turn to 5 small trials from China, we observe a possible suggestion of clinical improvement. An RCT comparing HCQ (400 mg daily) plus standard of care to standard of care alone in 62 COVID-19–positive patients with chest CT confirmation and mild illness (partial pressure arterial oxygen/fraction of inspired oxygen [PaO_2_/FiO_2_] >300) demonstrated a significantly quicker time to clinical recovery (defined as afebrile body temperature and resolution of cough) and improvement in chest CT imaging in the HCQ group.^5^ L. Chen et al corroborated a trend to shorter recovery with HCQ (200 mg twice daily) in a small study (n=48) of HCQ and standard of care vs CQ (500 to 1,000 mg daily) and standard of care vs standard of care.^8^ Only patients with mild or moderate illness (generally patients with oxygen saturation [SaO_2_] >93% and/or PaO_2_/FiO_2_ >300) were enrolled in these 2 studies. Standard of care was based on clinician judgment and was either not specified or varied widely to include antivirals, antibiotics, immunoglobulins, and/or corticosteroids.^5,8^ Thus, the generalizability of such studies to patients with severe COVID-19 who may need more aggressive intervention is questionable.

The 3 other small studies (n ranging from 30 to 150) from China were uniform in identifying a lack of significant difference in proportion or time to negative seroconversion in patients with confirmed COVID-19.^4,9,10^ Tang et al also observed no difference in clinical course, inflammatory markers, or mortality when HCQ 800 to 1,200 mg/day was added to standard of care therapy.^9^ Similarly, J. Chen et al and C. P. Chen et al observed no difference in mortality or side effects when HCQ 200 to 400 mg/day was added to standard-of-care therapy.^4,10^ These studies also primarily focused on patients with mild or moderate illness.

Turning back to trials outside of China, the potential drawbacks of HCQ and CQ regimens emerge. The Borba et al trial terminated prematurely because of the increased incidence of QTc interval prolongation and lethality in a high-dose (600 mg twice daily) CQ group.^11^ Boulware et al observed an increased risk of mild adverse events, including nausea (22.9% vs 7.7%) and diarrhea/abdominal discomfort (23.2% vs 4.3%), in the HCQ treatment group compared to the placebo group, a finding corroborated by the Mitjà et al study.^6,12^ The unblinded Mitjà et al trial had a high enrollment of health care workers (86.7% of study subjects), and 72.0% of patients taking HCQ reported adverse events vs 8.7% of patients in the control arm.^6^ Elevated aminotransferases were also noted as an adverse effect of HCQ in multiple trials and required discontinuation of the study drug in 1 patient in the J. Chen et al study.^4,7,8^ While these adverse events were mild in many cases, decreased adherence to HCQ compared to placebo was noted in 2 studies of HCQ use in outpatient populations; thus, mild adverse events—especially gastrointestinal symptoms including nausea, abdominal discomfort, and diarrhea—may affect the efficacy of HCQ treatment for asymptomatic patients or patients with low-acuity cases of COVID-19.^6,13^ Another consideration is that several RCTs specifically excluded patients with preexisting cardiac pathology, underlying QTc interval prolongation, or concomitant use of QTc-prolonging medications, therefore perhaps providing insufficient information about the deleterious cardiac outcomes of HCQ in the population at large.^6,8,10,12^

Confusion about what role, if any, HCQ should play in COVID-19 treatment is driven in part by significant study limitations, especially in terms of heterogeneous standard-of-care treatments and limited external validity. Themes that originated in the first studies from China were small sample sizes and skew of the patient populations to mild and moderate disease.^4,5,8-10^ More concerning from a methodology perspective were the frequent protocol deviations and the lack of placebo groups, control group treatment specifications, and blinding. We have summarized the limitations and challenges with the study designs for these 10 clinical trials in the Table. Ultimately, robust statistical understanding beyond simple *P* value dichotomy may be necessary to understand the nuances of and draw reasonable conclusions from underpowered trials.

## STUDY CHALLENGES DURING A PANDEMIC

COVID-19 has presented major challenges to the medical-academic community in terms of conducting clinical trials in an epidemiologically valid yet timely manner. From the studies presented here, we have determined that treatment with HCQ in patients with COVID-19 has not been shown to consistently improve clinical outcomes, although the majority of studies had significant design limitations. HCQ may not become part of the standard treatment for patients with COVID-19, but we can still glean lessons that can inform research in future pandemics. Even in the midst of a rapidly evolving pandemic, potential therapeutics should be rigorously tested. Although avenues for timely data dissemination should exist, the peer review process must continue to be held to a high standard and remain uninfluenced by political or personal conflicts of interest. Standard-of-care treatments used as comparisons should be truly standardized and specified in detail, even in preliminary scientific manuscripts. In addition, patient populations included in early studies must be chosen carefully; discussion of the utility of therapeutics that were only investigated in patients with mild or moderate illness must be heavily tempered when considering their use in patients with more serious disease. Further, the safety profile of novel interventions should be rigorously investigated in the general population. COVID-19 has provided fertile soil for the flourishing of clinical research, but both study designers and the reading audience must take great care to determine how the combined body of research ought to affect clinical care.
